# *In Vivo* Anti-Cancer Mechanism of Low-Molecular-Weight Fucosylated Chondroitin Sulfate (LFCS) from Sea Cucumber *Cucumaria frondosa*

**DOI:** 10.3390/molecules21050625

**Published:** 2016-05-12

**Authors:** Xiaoxiao Liu, Yong Liu, Jiejie Hao, Xiaoliang Zhao, Yinzhi Lang, Fei Fan, Chao Cai, Guoyun Li, Lijuan Zhang, Guangli Yu

**Affiliations:** 1 Key Laboratory of Marine Drugs, Ministry of Education, School of Medicine and Pharmacy, Ocean University of China, Qingdao 266003, China; sophielxx@163.com (X.L.); liuyong1990820@163.com (Y.L.); 2009haojie@ouc.edu.cn (J.H.); zhxl819@163.com (X.Z.); langyinzhi@163.com (Y.L.); fanfei203@163.com (F.F.); caic@ouc.edu.cn (C.C.); liguoyun808@126.com (G.L.); lijuanzhang@ouc.edu.cn (L.Z.); 2 Shandong Provincial Key Laboratory of Glycoscience and Glycoengineering, Ocean University of China, Qingdao 266003, China

**Keywords:** low-molecular-weight fucosylated chondroitin sulfate, anti-metastasis, anti-angiogenesis, Lewis lung carcinoma, mechanism, NF-κB

## Abstract

The low-molecular-weight fucosylated chondroitin sulfate (LFCS) was prepared from native fucosylated chondroitin sulfate (FCS), which was extracted and isolated from sea cucumber *Cucumaria frondosa*, and the anti-cancer mechanism of LFCS on mouse Lewis lung carcinoma (LLC) was investigated. The results showed that LFCS remarkably inhibited LLC growth and metastasis in a dose-dependent manner. LFCS induced cell cycle arrest by increasing p53/p21 expression and apoptosis through activation of caspase-3 activity in LLC cells. Meanwhile, LFCS suppressed the expression of vascular endothelial growth factor (VEGF), increased the expression of tissue inhibitor of metalloproteinase-1 (TIMP-1) and downregulated the matrix metalloproteinases (MMPs) level. Furthermore, LFCS significantly suppressed the activation of ERK1/2/p38 MAPK/NF-κB pathway, which played a prime role in expression of MMPs. All of these data indicate LFCS may be used as anti-cancer drug candidates and deserve further study.

## 1. Introduction

According to World Cancer Report 2014, cancer is the leading cause of mortality and contributes to more than 8.2 million deaths globally in 2012 [1]. Lung cancer remains the most common cancer with the highest incidence rate (12.9%) and mortality rate (19.4%) [2,3,4]. Non-small-cell lung cancer (NSCLC) accounts for 85% of all cases of lung cancer [5,6]. The high mortality rate is related to the low cure rate as a result of the metastasis, not the primary tumors [7,8].

Metastasis is responsible for 90% of mortality of cancer-associated disease [9]. Tumor metastasis is a complex multi-step process including uncontrolled proliferation of primary tumor, invasion, systemic dissemination, angiogenesis, and colonization. Unlimited proliferation is an acquired capability of cancer cells. p53 is a tumor suppressor transcription factor. Activation of p53 induces the expression of p21, which is a universal inhibitor of the cyclin-dependent kinases (Cdks), and cell cycle arrest [10,11,12]. Han *et al.* reported that p21 was required for senescence development of HCT116 cells treated with low concentrations of camptothecin [13]. Caspases work as essential mediators of apoptosis, and caspase-3 is a crucial trigger of apoptosis, which eventually leads to cell death [14,15]. Liu *et al.* reported that ellagic acid induced G0/G1 arrest through increased p53 level and induced apoptosis through activation of caspase-3 activity in human bladder cancer T24 cells [16]. To invade into the surrounding stroma, tumor cells need to degrade and breach the barrier of basement membranes and the extracellular matrix (ECM). Matrix metalloproteinases (MMPs) are the most important degrading enzymes [17]. MMP-9 is an important gelatinase to degrade ECM components especially Type IV collagen which is the major component of the basement membrane. Tissue inhibitor of metalloproteinase-1 (TIMP-1) is the natural inhibitor of MMP-9. Wu *et al.* reported that TIMP-1and MMP-9 may work as biomarkers to predict the progression and prognosis of breast cancer [18]. Angiogenesis primarily regulated by vascular endothelial growth factor (VEGF) is one of the critical steps in tumor growth and metastasis [19,20]. Huang *et al.* reported that fucoidan inhibited lung carcinoma metastasis by down-regulating expression of VEGF and MMPs [21]. Cancer metastasis is a multi-step process regulated by a complex signaling network [8]. The nuclear factor-kappa B (NF-κB) can induce the transcription, expression, and secretion of MMPs [22,23,24], which is regulated by mitogen-activated protein kinase (MAPKs) [25,26]. Three distinct MAPKs family members including extracellular signal-regulated kinase 1 and 2 (ERK1/2), c-Jun *N*-terminal kinase/stress-activated protein kinase (JNK/SAPK), and p38 play a major role in tumor progression and metastasis by inducing proteolytic enzymes that degrade the basement membrane, enhances cell migration [27,28]. Huang *et al.* reported that the expression and secretion of MMP-9 protein were induced through the activation of extracellular signal-regulated protein kinase (ERK) and nuclear factor-kB NF-κB signaling pathways [25].

Fucosylated chondroitin sulfate (FCS) is a heparin-like glycosaminoglycan (GAG) isolated from sea cucumbers. FCS is composed of a backbone consisted of repeating units of →4GlcAβ1→3GalNAcβ1→ with α-fucose branches linked to the *O*-3 position of GlcA residues. Recent studies have demonstrated that FCS possessed various biological activities, such as anti-coagulant [29], anti-thrombotic [30], and anti-viral effects [31]. Importantly, FCS showed a remarkable function in the inhibition of metastasis and thrombosis by NF-κB/tissue factor/factor Xa pathway in mouse melanoma B16F10 cell [32]. Additionally, FCS was able to suppress metastasis and inflammatory reaction by selectin blocking activity [33]. However, the application of native FCS resulted in an undesirable effect of platelet aggregation [34]. Several studies reported that LFCS exerted anti-thrombotic effect with less bleeding side effect [35], whereas there were scarcely reports about the anti-cancer activity and mechanism of LFCS.

In this study, we evaluated the anti-cancer activity of LFCS derived from sea cucumber *Cucumaria frondosa* using a mouse Lewis lung carcinoma model. The molecular mechanism of LFCS on tumor growth, invasion, metastasis, and angiogenesis were investigated as well. It is demonstrated that LFCS is a potential anti-tumor candidate capable of inhibiting tumor growth, metastasis, and angiogenesis by the MAPK (p38/ERK1/2)/NF-κB pathway.

## 2. Results and Discussion

### 2.1. LFCS Inhibits LLC Tumor Growth in Vivo

**Figure 1:**
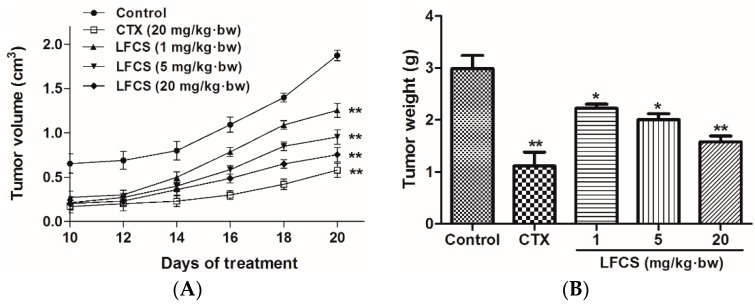
Anti-tumor effects of LFCS on Lewis lung cancer *in vivo*. LFCS significantly reduced the tumor volume and weight in C57BL/6 mice. (**A**) Tumor growth curves and (**B**) weight of the tumors. Mean weights of the tumors are 2.99 g, 1.12 g, 2.23 g, 2.00 g and 1.58 g, for the control group, CTX group, 1 mg/kg·bw, 5 mg/kg·bw, and 20 mg/kg·bw LFCS group, respectively. Data are represented as mean ± SD (*n* = 8). * *p* < 0.05, ** *p* < 0.01, significant difference compared with control group (NS group).

To study the antitumor activity of LFCS *in vivo*, we set up a mouse LLC metastasis model. A tumor growth curve was constructed as shown in Figure 1A. After 20 days of treatment, the mice were euthanized and the tumors were excised and weighed (Figure 1B). As shown in Figure 1, LFCS significantly reduced the tumor volume (*p* < 0.01) and weight in a dose-dependent manner *in vivo*. The inhibition rates of 1, 5, and 20 mg/kg·bw of LFCS were 25.8% (*p* < 0.05), 33.1% (*p* < 0.05), and 47.2% (*p* < 0.01), respectively.

### 2.2. LFCS Suppressed Lung Metastasis of LLC Cells In Vivo

**Figure 2:**
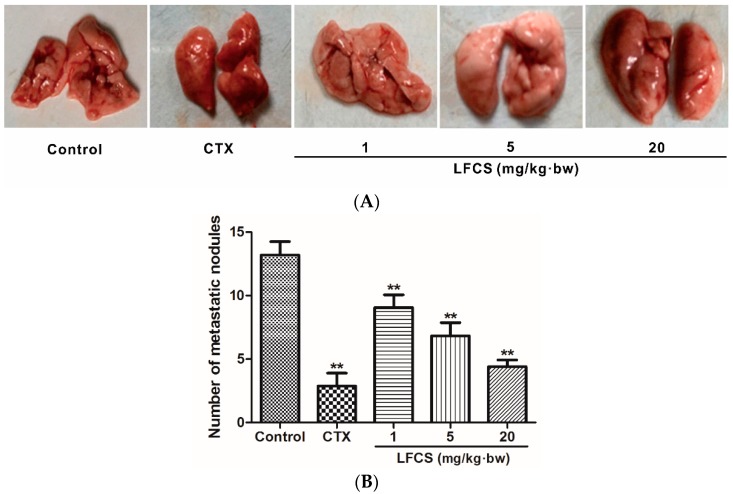
LFCS suppressed lung metastasis of LLC cells *in vivo*. (**A**) Representative photos of lungs with the metastatic colonies after treatment with LFCS or not. Tumor-bearing mice were euthanized, and the lungs excised and counted the metastatic nodules (**B**) Lung metastatic nodules. Data are represented as mean ± SD (*n* = 8). ** *p* < 0.01, significant difference compared with control group (NS group).

Cancer metastasis is the major cause of mortality in cancer patients. To investigate the anti-metastatic effect of LFCS on LLC cells *in vivo*, the tumor-bearing mice were euthanized, and the lungs excised and counted the metastatic nodules after LFCS treatment or not (Figure 2). Compared with the control group, LFCS significantly reduced the lung metastasis of LLC cells in dose-dependent manner (*p* < 0.01). The inhibition rates of 1, 5, and 20 mg/kg·bw of LFCS were 32.4%, 52.2%, and 69.1%, respectively.

### 2.3. LFCS Inhibited LLC Cell Proliferation and Cell Migration

**Figure 3:**
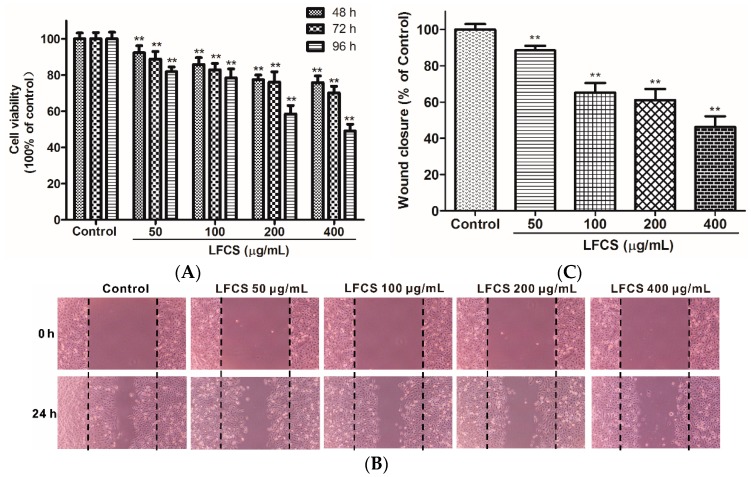
LFCS inhibited LLC cell proliferation and cell migration. (**A**) Inhibitory effect of LFCS on LLC cell proliferation. LLC cells were treated with 50, 100, 200, and 400 μg/mL LFCS separately for 48 h, 72 h and 96 h and measured for viability by MTT assay. Values relative to that of control group (in which cell viability is set as 100%) were shown; (**B**) inhibitory effect of LFCS on LLC cell migration. Representative photos of wound closures after treatment with LFCS or not (40× magnification). Black dotted lines indicate the wound edge; and (**C**) quantification of the effect of LFCS on LLC cell migration in the wound healing assay. Wound closure relative to that of control group (in which wound closure is set as 100%) was determined. Data were derived from three independent experiments and presented as mean ± SD. ** *p* < 0.01, significant difference compared with control group.

Clearly, the *in vivo* data described above demonstrated significant inhibitory effects of LFCS on tumor growth and metastasis. Since tumor cell proliferation and migration play critical roles in the process of tumor development [36,37], we then examined the effects of LFCS on LLC cell proliferation and migration by MTT assay and wound healing assay, respectively. For the MTT assay, LLC cells were incubated and treated with LFCS (50 μg/mL, 100 μg/mL, 200 μg/mL, and 400 μg/mL) for 48 h, 72 h, and 96 h. As shown in Figure 3A, LFCS decreased LLC cell viability in dose- and time-dependent manners (*p* < 0.01). After treatment for 96 h, 400 μg/mL LFCS decreased viable LLC cell number by 50.9% as compared with untreated control group. In the wound healing assay, the chemotactic motility of LLC cells were investigated after treatment with or without LFCS (50 μg/mL, 100 μg/mL, 200 μg/mL, and 400 μg/mL) for 24 h. As shown in Figure 3B, untreated cells migrated into the wounded area of the cell monolayer, whereas LFCS remarkably inhibited the LLC cells migration in a dose-dependent manner. The inhibition effects were measured to be 11.5%, 34.7%, 38.8%, and 53.7% for 50, 100, 200, and 400 μg/mL LFCS, respectively (Figure 3C).

### 2.4. LFCS Suppressed Proliferation and Migration of HUVEC

**Figure 4:**
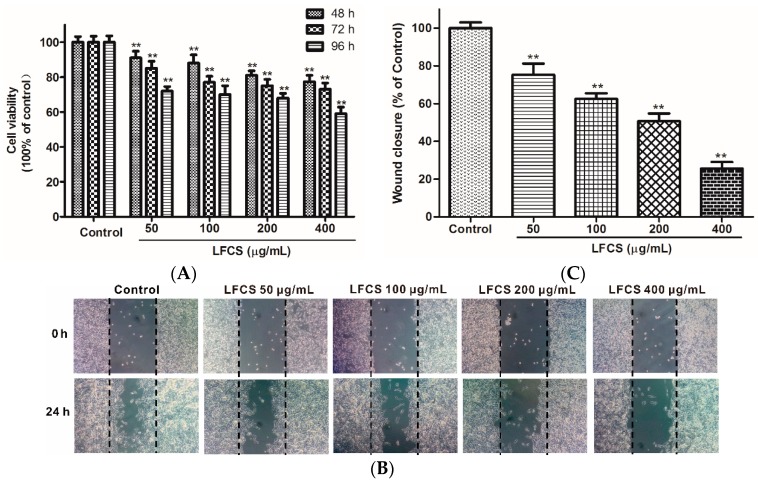
LFCS suppressed proliferation and cell migration of HUVEC. (**A**) Inhibitory effect of LFCS on HUVEC proliferation. HUVEC were treated with 50, 100, 200, and 400 μg/mL LFCS separately for 48 h, 72 h, and 96 h and measured for viability by MTT assay. Values relative to that of control group (in which cell viability is set as 100%) were shown; (**B**) inhibitory effect of LFCS on HUVEC migration. Representative photos of wound closures after treatment with LFCS or not (10× magnification); and (**C**) quantification of the effect of LFCS on HUVEC migration. Wound closure relative to that of control group (in which wound closure is set as 100%). Data were derived from three independent experiments and presented as mean ± SD. ** *p* < 0.01, significant difference compared with control group.

Angiogenesis plays an important role in the growth, progression, and metastasis of a tumor by providing oxygen and nutrients to the tumor cells and also removing waste products, whereas endothelial cell migration is essential for angiogenesis [38]. LFCS demonstrated significant anti-tumor efficacy in LLC-bearing mice, and we next examined whether LFCS could affect angiogenesis by investigating its effects on human umbilical vein endothelial cells (HUVEC) migration using wound healing assays. The chemotactic motility of HUVEC were investigated after treatment with or without LFCS (50 μg/mL, 100 μg/mL, 200 μg/mL, and 400 μg/mL) for 24 h. As shown in Figure 4A, cells in control group migrated into the wounded area of the cell monolayer, whereas HUVEC with LFCS treatment migrated remarkably slowly. It demonstrated that LFCS inhibited HUVEC migration in a dose-dependent manner by 24.8%, 37.5%, 49.3%, and 74.4% for 50, 100, 200, and 400 μg/mL LFCS, respectively (Figure 4B). Then, we determined the viability of HUVEC by a MTT assay to assess whether the inhibitory ability of LFCS on HUVEC migration was a result of the inhibition on HUVEC proliferation. HUVEC were treated with 50, 100, 200, and 400 μg/mL LFCS for 48 h, 72 h and 96 h separately. As shown in Figure 4C, LFCS reduced HUVEC viability in dose- and time-dependent manners (*p* < 0.01). After treatment for 96 h, 400 μg/mL LFCS decreased viable HUVEC number by 41.0% as compared with vehicle control. Thus, LFCS inhibited angiogenesis by suppressing migration and proliferation of endothelial cells.

### 2.5. LFCS Induced Cell Cycle Arrest and Apoptosis by Uprgulating Protein Expression of p53, p21, and Caspase-3

**Figure 5:**
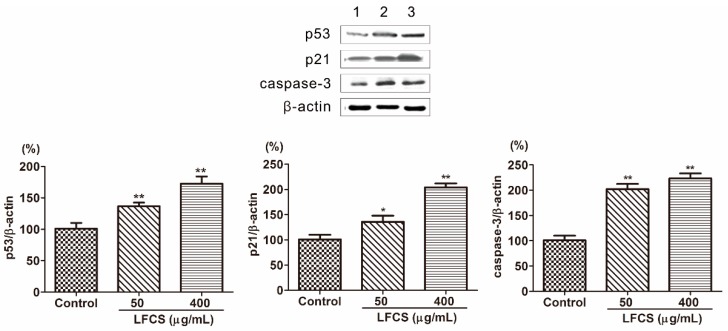
LFCS increased protein expression of p53, p21, and caspase-3 in LLC cells after 72 h treatment of LFCS detected by Western blot. Expression of the β-actin was used as internal control. Lane 1: vehicle-treated cells; Lane 2: 50 μg/mL LFCS-treated cells; Lane 3: 400 μg/mL LFCS-treated cells. Data were presented as mean ± SD and derived from one representative experiment performed in triplicate. * *p* < 0.05, ** *p* < 0.01, significant difference compared with control group.

Tumor occurrence is a result of deviant of cell proliferation, differentiation, and apoptosis. Tumors display six essential characteristics of self-sufficiency in growth signals, insensitivity to antigrowth signals, evasion of apoptosis, limitless replicative potential, sustained angiogenesis and tissue invasion, and metastasis [36,39]. To investigate the mechanism that LFCS inhibits tumor growth, metastasis, and angiogenesis, we examined protein expression in the molecular pathways induced by LFCS. p53 is a tumor suppressor transcription factor, and induces transcription of p21 and inhibition of cyclin-dependent kinases by p21 resulting in cell cycle arrest [10,11,12]. As shown in Figure 5, the expression of p53 and p21 were significantly increased by LFCS (Figure 5). The result indicated that LFCS increased the expression of p53 to upregulate the expression of p21 resulting in cell cycle arrest. Apoptosis is a process of programmed cell death requiring activation of several signaling cascades. Caspases are essential mediators of apoptosis, and caspase-3 is crucial for cell death and some certain biochemical events associated with apoptosis [14,15]. After LFCS treatment, the level of caspase-3 increased (Figure 5) and demonstrated that LFCS induced a caspase-3-dependent apoptotic pathway in LLC cells consistent with effect of fucoidan on breast cancer [40].

### 2.6. LFCS Suppressed Metastasis and Angiogenesis by Regulating Protein Expression of MMP-9, TIMP-1, VEGF, p38, ERK1/2, and NF-κB

**Figure 6:**
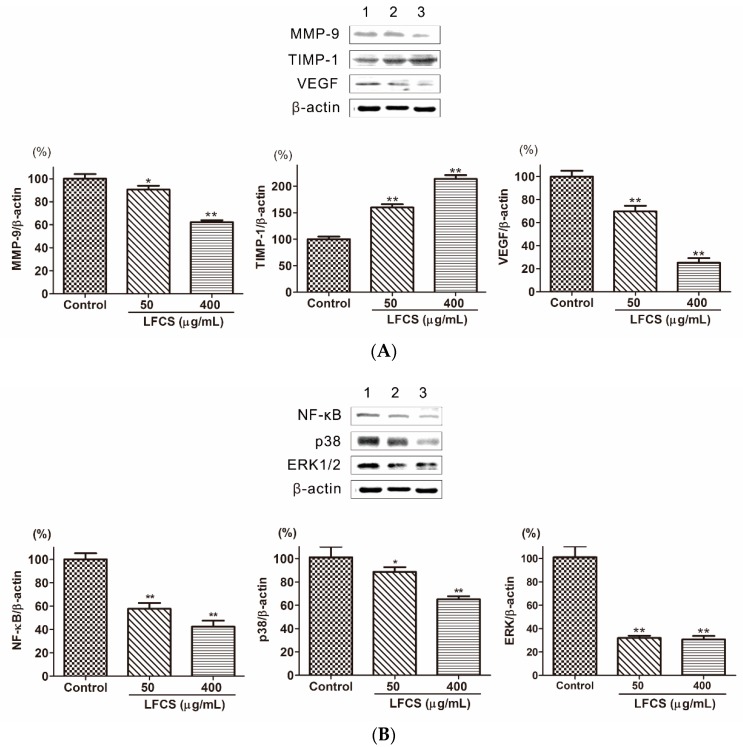
LFCS suppressed metastasis and angiogenesis by regulating protein expression of MMP-9, TIMP-1, VEGF, p38, ERK1/2, and NF-κB in LLC cells after 72 h treatment of LFCS detected by Western blot. (**A**) LFCS increased TIMP-1, and decreased MMP-9 and VEGF expression; (**B**) LFCS suppressed NF-κB, p38 and ERK1/2 expression. Expression of the β-actin was used as internal control. Lane 1: vehicle-treated cells; Lane 2: 50 μg/mL LFCS-treated cells; Lane 3: 400 μg/mL LFCS-treated cells. Data were presented as mean ± SD and derived from one representative experiment performed in triplicate. * *p* < 0.05, ** *p* < 0.01, significant difference compared with control group.

Cancer metastasis is a multi-step process regulated by a complex signaling network [8]. In order to invade into the surrounding stroma, tumor cells need to breach the basement membrane barrier by secreting proteinases to degrade the extracellular matrix (ECM). Matrix metalloproteinases (MMPs) are the most important degrading enzymes [17]. MMP-9 is an important gelatinase to degrade ECM components especially Type IV collagen which is the major component of the basement membrane. As shown in Figure 6A, the level of MMP-9 was decreased in LLC cells treated with LFCS. Tissue inhibitor of metalloproteinase-1 (TIMP-1) is the natural inhibitor of MMP-9, and it can suppress MMP-9 to inhibit tumor cell invasion and migration [18]. Then we determined the protein level of TIMP-1 by Western blot to assess whether the inhibitory ability of LFCS on MMP-9 protein expression was a result of the inhibition on TIMP-1. The result showed that LFCS up-regulated TIMP-1 level in LLC cells (Figure 6A). Thus LFCS inhibited degradation effect of MMP-9 by directly decreasing expression of MMP-9 and indirectly increasing level of TIMP-1. Angiogenesis plays a critical role in tumor growth and metastasis. The vascular endothelial growth factor (VEGF) is one of representative proangiogenic factors and promotes vascular endothelial growth and mitosis to form vascular plexus [41]. To investigate whether LFCS suppresses the expression of VEGF to inhibit angiogenesis, we examined the VEGF expression in LLC cells after treatment with LFCS. As shown in Figure 6A, the level of VEGF increased. The result demonstrated that LFCS inhibited tumor metastasis by increasing TIMP-1 and decreasing MMP-9 expression, and suppressing VEGF to inhibit angiogenesis.

Tumor metastasis is a complex multistep process modulated by signaling pathways. The nuclear factor-kappa B (NF-κB) is highly activated in diverse cancers and plays a critical role in tumor cell proliferation, angiogenesis, and metastasis, resulting in aggressiveness of tumors [42]. NF-κB can induce transcription, expression, and secretion of MMPs [22,23,24], and this progress is regulated by mitogen-activated protein kinase (MAPKs) [25,26]. Three distinct MAPKs family members, including extracellular signal-regulated kinase 1 and 2 (ERK1/2), c-Jun N-terminal kinase/stress-activated protein kinase (JNK/SAPK), and p38, play major roles in tumor progression and metastasis by inducing proteolytic enzymes that degrade the basement membrane, enhances cell migration [27,28]. The expression and secretion of MMP-9 protein were induced through the activation of extracellular signal-regulated protein kinase (ERK) and nuclear factor kappa B (NF-κB) signaling pathways [24]. To elucidate whether LFCS regulates these signaling pathways in LLC cells, we examined the expression of NF-κB, p38 and ERK1/2 in LLC cells after treatment with LFCS. As shown in Figure 6B, the level of NF-κB, p38 and ERK1/2 were decreased by LFCS. The result demonstrated that LFCS inhibited tumor metastasis by activated MAPKs (p38 and ERK1/2)/NF-κB pathway.

## 3. Materials and Methods

### 3.1. Materials

The low-molecular-weight fucosylated chondroitin sulfate (LFCS) was a depolymerized fragment of native fucosylated chondroitin sulfate (FCS), which was isolated from the body walls of the sea cucumbers *C. frondosa* purchased in the Nanshan market of Qingdao City, China. LFCS contained GlcA, GalN, Fuc, and sulfate in molar ratios of 1.0:1.0:1.1:3.2 with a molecular weight of 12.0 kDa. LFCS mainly composed of a chondroitin sulfate-like backbone of →4GlcAβ1→3GalNAc6Sβ1→ with sulfated α-Fuc branches linked to the *O*-3 position of GlcA residues was characterized by nuclear magnetic resonance (NMR) analysis (Figure S1). Normal salt (NS) was purchased from Cisen Pharmaceutical Co., Ltd. (Jining, Shandong, China). Cyclophosphamide for injection (CTX) was purchased from Hengrui Medicine Co., Ltd. (Lianyungang, Jiangsu, China). 5-diphenyl tetrazolium bromide (MTT) was purchased from Sigma-Aldrich (St. Louis, MO, USA). Anti-p53, p21, caspase-3, MMP-9, TIMP-1, VEGF, p38, ERK1/2, NF-κB, and β-actin antibodies were purchased from Santa Cruz Biotechnology (Santa Cruz, CA, USA). All other chemicals and solvents used were of analytical grade unless otherwise specified.

### 3.2. Cells and Cell Culture

Lewis lung carcinoma cells (LLC) and human umbilical vein endothelial cells (HUVECs) were purchased from the Cell Bank of the Type Culture Collection Center of the Chinese Academy of Sciences in Shanghai, China. These cells were maintained in DMEM culture medium (Gino Biological Medicine Technology Co., Ltd., Hangzhou, China) supplemented with 10% fetal bovine serum (FBS) (*v*/*v*), 100 U/mL penicillin, and 100 μg/mL streptomycin, and cultured in an incubator at 37 °C under a humidified atmosphere of 5% CO_2_.

### 3.3. Animals and Ethical Approval

Male C57BL/6 mice (6–8 weeks old) were purchased from Experimental Animals and Animal Experiments Center (Qingdao, China). The animals were maintained in a climate controlled room (12:12 dark-light cycle with a constant room temperature of 21 ± 1 °C) and fed standard rodent chow and water *ad libitum*. Mice were acclimatized for at least one week. All experiments were performed in accordance to internationally-accepted guidelines on laboratory animal use.

### 3.4. In Vivo LLC Cells Metastasis Model

To study the antitumor activity of LFCS *in vivo*, approximately 2 × 10^6^ log growth-phase LLC cells in 0.2 mL NS were injected through subcutaneous injection to the right armpit of male C57BL/6 mice on day one. One day after the tumor cell inoculation, a volume of 0.2 mL of NS (as blank control), CTX (20 mg/kg, as a positive reference) and LFCS (1, 5 and 20 mg/kg) was administered intraperitoneally to the mice (*n* = 8) once a day for 20 days, respectively. After 10 days, tumors were visible at the inoculated sites, and their sizes were measured in two perpendicular dimensions (a = length, b = width) with a vernier caliper and recorded as a volume (cm^3^) as calculated by a × b^2^/2 every two days. Then a tumor growth curve was constructed. After 20 days of treatment, the mice were euthanized, and the lungs and tumors were excised and weighted. The number of lung metastatic foci was counted.

### 3.5. Cell Proliferation Assays

Cell proliferation was measured by using the MTT assay based on the ability of viable cells to change from soluble yellow tetrazolium salt to blue formazan crystals. Briefly, LLC cells/HUVECs (1 × 10^4^ cells/mL) were seeded in 96-well plates and treated with or without LFCS (50 μg/mL, 100 μg/mL, 200 μg/mL, and 400 μg/mL). After 48 h, 72 h and 96 h, the cells were treated with 20 μL MTT (5 mg/mL) and re-incubated for 4 h. After removal of the supernatant, 150 μL of DMSO was added to dissolve the blue crystals. The absorbance was recorded at 490 nm by a Bio-Tek Elx 808 microplate reader (BioTek China Shanghai Office, Shanghai, China).

### 3.6. Cell Migration Assays

Cell migration was determined using the wound healing assays. Briefly, LLC cells/HUVECs (1 × 10^5^ cells/well) were seeded in 24-well plates and grown as monolayer cells at 90%–95% confluence. Then the monolayer was carefully wounded by sterile pipette tips (*t* = 0 h). After removal of the detached cells, the cells were treated with or without LFCS (50 μg/mL, 100 μg/mL, 200 μg/mL and 400 μg/mL) for 24 h and photographed by a microscope (Olympus, CKX41, Tokyo, Japan) immediately (*t* = 24 h).

### 3.7. Western Blot Analysis

LLC cells were seeded in six-well plates and treated with LFCS (50 μg/mL and 400 μg/mL) for 72 h. After removal of the supernatant, the cells were washed twice with ice-cold PBS and lysed in 50 μL RIPA lysis buffer (Beyotime Institute of Biotechnology, Beijing, China) on ice for 30 min with sonication for 10 s every 10 min. Protein concentrations were measured using a BCA protein assay kit (Beyotime Institute of Biotechnology, Beijing, China). Equal amounts of protein were fractionated by SDS-PAGE and then transferred to PVDF membranes. After blocking with 5% non-fat milk in TBST buffer for 1 h, the membranes were incubated with various primary antibodies against p53, p21, caspase-3, MMP-1, TIMP-1, VEGF, p38, ERK1/2, NF-κB, and β-actin at 4 °C overnight. After washing, the membranes were incubated with alkaline phosphatase-labeled secondary antibodies (Boster, Wuhan, China) for 2 h. The proteins were then detected using a BCIP/NBT Alkaline Phosphatase Color Development Kit (Beyotime Institute of Biotechnology, Beijing, China).

### 3.8. Statistical Analysis

Results were expressed as mean ± SD. Data were analyzed by ANOVA and statistical significance was considered at *p* < 0.05 in all cases.

## 4. Conclusions

In this study, LFCS was demonstrated to have remarkable inhibition on Lewis lung carcinoma growth and metastasis in a dose-dependent manner *in vivo*. The anticancer activity was related to p53/p21-induced cell cycle arrest, caspase-3-induced apoptosis, VEGF-mediated angiogenesis, and TIMP/MMPs-mediated metastasis by the ERK1/2/p38 MAPK/NF-κB pathway. Our studies suggest that LFCS may be an anti-tumor drug candidate that deserves further research.

## Supplementary Materials

Supplementary materials can be accessed at: http://www.mdpi.com/1420-3049/21/5/625/s1.
