# Supplementary material for: In Vivo Anti-Cancer Mechanism of Low-Molecular-Weight Fucosylated Chondroitin Sulfate (LFCS) from Sea Cucumber Cucumaria frondosa
Source: Molecules. 2016 May 12;21(5):625. doi: 10.3390/molecules21050625 (PMC6273849; doi:10.3390/molecules21050625)
Supplement: Supplementary file 1 [file molecules-21-00625-s001.pdf]

Supplementary Materials: *In Vivo* Anti-Cancer Mechanism of Low-Molecular-Weight Fucosylated Chondroitin Sulfate (LFCS) from Sea Cucumber *Cucumaria frondosa*

Xiaoxiao Liu, Yong Liu, Jiejie Hao, Xiaoliang Zhao, Yinzhi Lang, Fei Fan, Chao Cai, Guoyun Li, Lijuan Zhang and Guangli Yu

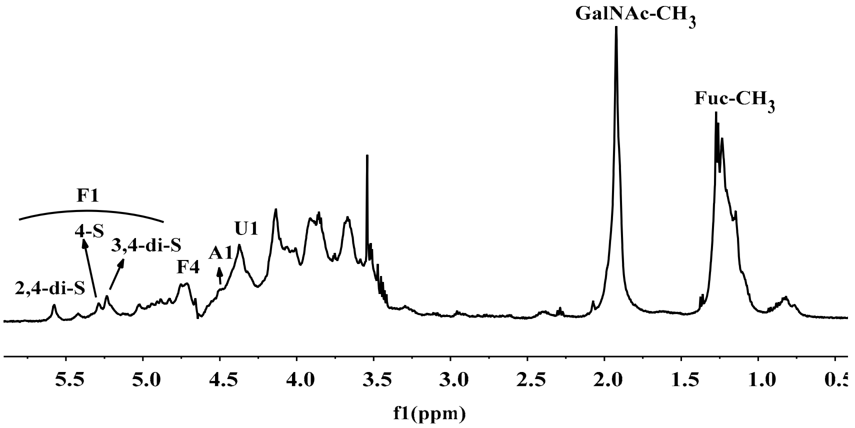

(**A**)

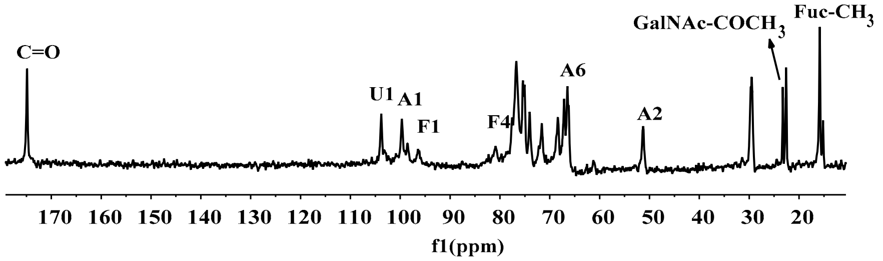

(**B**)

Figure S1. NMR spectra of LFCS. (**A**) ^{1}H-NMR spectrum of LFCS. 4-S, 4-O-sulfated fucose; 2,4-di-S, 2,4-disulfated fucose; 3,4-di-S, 3,4-disulfated fucose; F1, H1 signal of fucose; F4, H4 signal of fucose; A1, H1 signal of GalNAc; U1, H1 signal of GlcA; and (**B**) ^{13}C-NMR spectrum of LFCS. F1, C1 signal of fucose; F4, C4 signal of fucose; A1, C1 signal of GalNAc; A2, C2 signal of GalNAc; A6, C6 signal of GalNAc6S; U1, C1 signal of GlcA.
